# Research into an Association between Anhedonia and Decreased REM Latency in Moderately to Severely Depressed Patients

**DOI:** 10.1155/2018/1636574

**Published:** 2018-07-02

**Authors:** Laurie Nizet, Xavier Montana, Jean-Pol Lanquart, Gwenolé Loas

**Affiliations:** Department of Psychiatry and Sleep Laboratory, Erasmus Hospital, ULB, Brussels, Belgium

## Abstract

Anhedonia stands as a core symptom and potential trait marker of major depressive disorder (MDD). The importance of rapid eye movement sleep latency (REML) as a biological marker of depression has previously and repeatedly been studied. The aim of this paper is to analyse the relationship between anhedonia and REML in moderately to severely depressed patients. The shortened Beck Depression Inventory (BDI-13) was chosen to assess depressive symptoms and, among them, more particularly, anhedonic symptoms. Two-way ANCOVA was used for statistical analyses. A significant association between anhedonic symptoms and REML was found when the number of sleep cycles (NCy) and the severity of depression were added as covariates. Our findings suggest that REML may be a useful variable to differentiate some diagnostic subtypes of depression related to anhedonia.

## 1. Introduction

Major depressive disorder (MDD) is associated with increased percentages of invalidity, morbidity, and mortality. The World Health Organization has ranked it as one of the most burdensome diseases in the world [1].

In DSM-V, MDD's diagnosis involves at least one of the two following core symptoms: depressed mood or anhedonia known as the loss of interest or pleasure in daily activities.

It has been theorized by Clark and Watson in their tripartite model of depression and anxiety that anhedonia is a specific manifestation of depression [2].

According to Loas, the symptom of anhedonia appears as a potential inherent trait marker of MDD [3].

Some authors have demonstrated the specificity of anhedonia in mood disorder compared to anxiety disorder [4] and compared to schizophrenia [5].

Fawcett et al. have supported the idea that anhedonia is a qualitative factor of an “endogenomorphic” or “melancholic” subtype of depression [6].

This concept of depressive subtypes, considered for several years, is upheld by Fletcher et al.'s [7]. Their work also puts anhedonia in a central position in the search of these subtypes' delimitation.

Disturbed sleep is reported by depressive patients up to 90% [8].

Previous research has shown that some EEG sleep features may represent a biomarker of MDD [9].

Reviewing the literature, we found that very few studies analysed the connections between sleep architecture and specific depressive symptoms.

Baglioni et al. have already suggested that different REM sleep alterations could distinguish specific symptomatology in mental disorders [10].

The latency of rapid eye movement sleep (REML) is considered to be one of the main objective sleep parameters. It is defined as the time between sleep onset and the first REM sleep period.

REML has drawn extensive consideration in psychiatric research since the association between a shortened value and the diagnosis of severe MDD was established by Kupfer and Forster in 1972 [11].

REML is thought to be the most specific quantitative biological marker separating depressive patients from healthy subjects but also from other psychiatric disorders as shown in the meta-analysis by Benca et al. [12] and more recently by Asaad et al. in a study comparing the polysomnographic characteristics of bipolar hypomanic patients with unipolar depressed patients [13].

It has even been assumed that shortened REML might be a clue to classify the depressive disorders. Indeed, the REM sleep disturbances have been showed to be positively correlated with MDD severity and duration [11, 14, 15].

The aim of the paper is to test the hypothesis, for the first time, that moderately to severely depressed subjects presenting with high anhedonia have shortened REML comparatively to moderately to severely depressed subjects presenting with low anhedonia.

The comparison of the two depressed groups will take into account the potential confounding variables and notably, gender, age, depressive severity, and number of cycles.

## 2. Material and Methods

### 2.1. Database, Inclusion, and Exclusion Criteria

The Ethics Committee of Erasmus Hospital gave us a favorable opinion for this study (Protocol P2015/236, Brussels, May 19, 2015).

Our sleep laboratory database contained 3511 subjects aged over 18 years having undergone a full night of polysomnography between 2003 and 2014 in response to a sleeping complaint.

This sample excluded patients suffering from narcolepsy and schizophrenia, as well as those presenting a history of drug use or alcoholism.

The Apnea-Hypopnea Index threshold was set strictly above 4 per minute and the 1510 subjects above this value were excluded from the sample because of the well-documented effect of sleep apnea-hypopnea syndrome on sleep architecture and fragmentation [16].

All subjects were asked to complete the shortened Beck Depression Inventory (BDI-13), a solid and well-validated measure of depressive symptomatology [17]. Only 1666 subjects correctly filled the BDI-13 and were thus included in the study.

As our work is based on a register study, patient treatments could not be controlled because this data was not available.

The alpha Cronbach of the BDI-13 was 0.88 with a mean interitem correlation of 0.37. The alpha Cronbach of the anhedonia subscale (items D, H, and K) of the BDI-13 was 0.64 with a mean interitem correlation of 0.38. The alpha Cronbach of the BDI-11 (without the items D, H, and K) was 0.85 with a mean interitem correlation of 0.37.

### 2.2. Sleep Recording and Scoring

Polysomnography was recorded with a 19-channel digital polygraph (Brainnet™, MEDATEC, Brussels, Belgium). Two electrooculograms (EOG), three electroencephalograms (Fz-Ax, Cz-Ax, and Oz-Ax, where Ax is a mastoid reference), one submental electromyogram (EMG), and electrocardiographic activity (EGC) were recorded. Oxyhemoglobin saturation was measured using pulse-oximetry (Biox 3740™, OHMEDA, Louisville, CO). Oro-nasal airflow was detected with thermistors (Infinity™, Sleepmate Technologies, Midlothian, VA). Thoracic and abdominal respiratory movements were recorded with piezoelectric sensors (Resp-EZ™, Sleepmate Technologies, Midlothian, VA). Leg movements were detected with ankle piezoelectric movements strain gauges (Moving Images™, Sleepmate Technologies, Midlothian, VA).

To eliminate low frequency artefacts, drift, and offsets, the following time constants were set in the Brainnet polygraph: 0.3 s for the EEG and 1 s for the EOG. Before sampling, the signals were filtered through a low-pass antialiasing analogue filter, with a cut-off frequency of 35 Hz. All channels were sampled at 200 Hz.

Respiratory sound was recorded with a microphone (MKE™, Sennheiser, Wedemark, Germany) inserted into a stethoscope fixed to the larynx. The sound was sampled at 2000 Hz and a rectified sound envelop was also sampled at 50 Hz. This technique allows the visual display of the sound intensity and the EEG-synchronised audio replay through headphones.

The Brainnet polygraph samples the signals on 12 bits and sends the resulting data to an Ethernet network, via the Netbios protocol. An acquisition program has been developed (Endymion 1993-2017, Sleep Laboratory, Erasme Hospital) to read and store the data in the EDF file format [18]. For subsequent analysis, EEGs were stored at 100 Hz, the EOG at 50 Hz, and the ECG at 200 Hz. To avoid aliasing, appropriate low-pass filters were applied before subsampling.

All subsequent analyses, such as stage determination, spectrum calculation, and heart rate analysis, were carried out on the sampled data, avoiding synchronisation problems between the stages and the other calculations.

Recordings were visually scored by specialized technicians to determine the different stages of sleep as classified by Rechtschaffen and Kales (interjudge agreement score of 85%) [19].

### 2.3. Study Design

**Figure 1:**
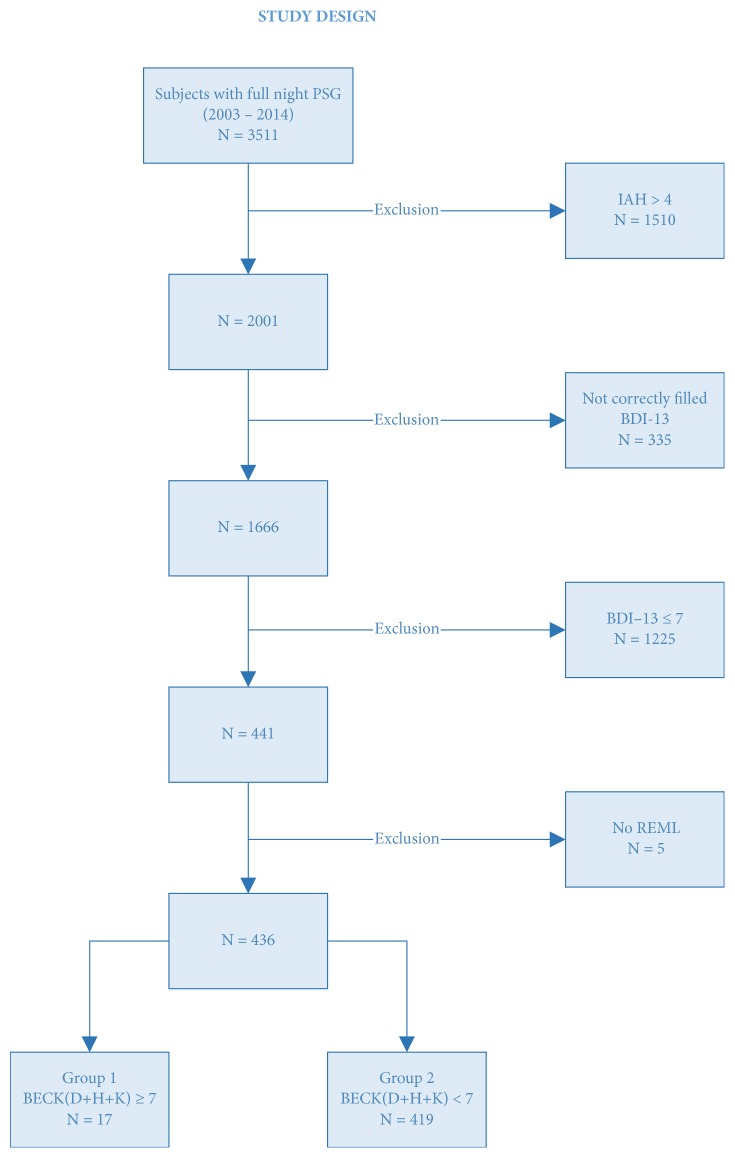


We decided to focus on the moderately to highly depressed patients (see Figure 1).

Thus, 1225 subjects were excluded from the sample because of their score below 7 at the BDI-13.

We had to remove 5 subjects because of their lack of REM sleep.

Anhedonia was rated using the anhedonia subscale of the BDI as used by Winer et al. [20]. The short form of the BDI includes 3 items of the original version that can be used to rate anhedonia: item # D (lack of satisfaction), item # H (social withdrawal or loss of interest), and item # K (work inhibition).

The remaining moderately to highly depressed subjects were divided into two groups according to their anhedonic score: 17 subjects in group 1 (anhedonic, (D+H+K) >=7) and 419 subjects in group 2 (nonanhedonic, (D+H+K) < 7).

### 2.4. Statistics

Normal distribution of our variables according to group size was assessed using Kolmogorov-Smirnov test. The distribution of our dependent variable (REML) in our sample was not normally distributed and was thus modified by a log10 transformation.

Two-way ANCOVA (parametric-test) was used for statistical analysis.

As group comparison showed significant differences for the number of cycles (NCy) and the BDI-10 scores (without the D, H, and K items), those variables were used as covariates in our model.

Age was controlled as another covariate and gender as a main factor. Indeed, according to Reynolds et al., significant age and gender effects are demonstrated for REML among other sleep variables [21].

The statistical software used for all analysis was IBM SPSS version 23.

## 3. Results

The result of the two-way ANCOVA comparing REML and gender in our two groups while introducing NCy, BDI-10 scores, and age as covariates showed a significant difference (F(5,429)=69.91; p<0.005), with a lower value in the anhedonic group.

See Table 1.

## 4. Discussion

The findings of this study indicate that a shortened REML is significantly associated with anhedonic symptoms in moderately to highly depressed patients when controlled by NCy and by severity of the depressive symptoms.

As several past studies have been omitted to control biases related to REML, the strength of our work resides in the adjustment of our dependent variable (REML) for gender, age, depression severity, and the number of cycles.

In a research on healthy subjects, Le Bon et al. demonstrated a strong inverse correlation between NCy and REML, recommending that NCy should be added as a covariate when sleep variables connected to cycles were studied [22].

In a further publication on MDD outpatients, Le Bon et al. showed the same inverse correlation and suggested that a shortened REML, as found in MDD and other psychiatric disorders, would imply a greater number of cycles [23].

With regard to MDD patients, Merica et al. had previously reported more sleep cycles in comparison to controls [24]. Our experiments are in line with these previous findings.

Anhedonia is one of the two core symptoms of depression and is highly connected to the severity of the depressive episode. The presence of anhedonia was found to be one of the risk factors predicting severity illness in MDD [25].

Perlis et al. research showed a relationship between sleep EEG variables disturbance and the core features of depression [26].

Preliminary work in this field has already established that, among other endogenous symptoms of depression, pervasive anhedonia is associated with reduced REML [27].

Furthermore, the inability to experience pleasure can be induced in animals by a chronic stress regimen which results in a reduction of sucrose consumption, a symptom equivalent to anhedonia according to Willner [28, 29]. This chronic mild stress animal model of depression was substantiated since then by different authors as Gronli et al. [30].

Several experimental studies carried out on animals have reported an association between sleep disturbances, including shortened REML, and reduced sucrose intake [31–34].

These findings lend support to our present results.

Interestingly, one study achieved on mice drew our attention to a correlation between a particular mouse phenotype (helpless) and alterations of sleep patterns but also anhedonia [35]. This suggests a future interest in the potential examination of genotype aspects of vulnerability to anhedonic symptoms and more generally to depression.

We are aware that our research may have a number of limitations.

To begin with, a possible source of error is that we were not able to exhaustively go through all the psychoactive drugs taken by our subjects. This major data was not available in the register. It is more than plausible that it might have influenced both sleep variables and responses to the shortened BDI-13.

Indeed, in their review of 2015, Torterolo et al. emphasized the implication of the serotoninergic, GABAergic, and MCHergic (melatonin-concentrating hormone) systems in REM sleep [36]. Furthermore, Schmidt et al. demonstrated that scores in BDI-II and serum levels of MCH significantly decreased during antidepressive treatment [37]. However, we could consider our database as a representative random sample in regard of eventual treatments due to the large number of subjects.

Inevitably, a concern appears with the questionable legitimacy in setting apart the anhedonia items from the other BDI-13 items. Joiner et al. have already used this method in previous research and have stated that the anhedonic subscale, while not being validated, can offer us an initial observation of anhedonia in depressed patients [5].

Surprisingly, REML values in the MDD patients were higher than we expected, appearing to be in the same range as the literature considering normal REML [38, 39].

A downside factor regarding our methodology is that the two groups were significantly unbalanced (N1=17 versus N2=419). Nevertheless, to the best of our knowledge, the relationship between anhedonia and REML controlled by NCy has not been investigated in MDD patients to date. Further studies must be conducted to better formalize the association between these variables.

In their meta-analysis, Benca and his coworkers have previously raised the issue of the analysis of subtypes of depression [12]. According to them, whether shortened REML could be expected in certain subgroups of patients with particular symptom clusters had to be determined. This is in good agreement with our findings that support the idea of a possible clinical dichotomy separating anhedonic versus nonanhedonic depression.

Therefore, further research focusing on this topic is required and needs to be performed with a well-developed scale of anhedonia to assess whether our present results can be supported.

As shown in a clinical review of 2013 [40], REM sleep dysregulation in depression has proved to be valuable and robust from clinical and research perspectives and thus deserves more extended exploration.

Our study design utilised REML as a continuous variable to analyse its connection to anhedonia. Further research could consider examining the association between REML and anhedonic symptoms in depressed patients by cutting off the REML variable in predetermined thresholds to determine more precisely the shortened duration of REML.

## 5. Conclusion

This paper has highlighted the significant association between a shortened REML and the major symptom of anhedonia in patients suffering from moderate to severe MDD when age, gender, the number of sleep cycles, and the severity of MDD were taken into account.

Our work had led us to conclude that understanding the relationship that underlies specific core symptoms of depression and sleep variables may be decisive for the improvement of diagnostic criteria and for a more accurate delimitation of depressive subtypes.

## Figures and Tables

**Table 1:** 

Descriptive REML groups	
**Group 1**	

N	17

Mean (min)	84.726

SD (min)	44.964

**Group 2**	

N	419

Mean (min)	125.810

SD (min)	87.235

## Data Availability

The database used to support the findings of this study is restricted by the Ethics Committee of Erasmus Hospital in order to protect patient privacy. Data are available from Dr. Nizet Laurie and Dr. Hein Matthieu (Department of Psychiatry and Sleep Laboratory, Erasmus Hospital) for researchers who meet the criteria for access to confidential data.

## References

[1] World Health Organization. (2015). *Depression Fact Sheet*.

[2] Clark L. A., Watson D. (1991). Tripartite model of anxiety and depression: psychometric evidence and taxonomic implications. *Journal of Abnormal Psychology*.

[3] Loas G. (1996). Vulnerability to depression: A model centered on anhedonia. *Journal of Affective Disorders*.

[4] Watson D., Clark L. A., Carey G. (1988). Positive and negative affectivity and their relation to anxiety and depressive disorders. *Journal of Abnormal Psychology*.

[5] Joiner T. E., Brown J. S., Metalsky G. I. (2003). A test of the tripartite model's prediction of anhedonia's specificity to depression: Patients with major depression versus patients with schizophrenia. *Psychiatry Research*.

[6] Fawcett J., Clark D. C., Scheftner W. A. (1983). Assessing anhedonia in psychiatric patients. *Archives of General Psychiatry*.

[7] Fletcher K., Parker G., Paterson A., Fava M., Iosifescu D., Pizzagalli D. A. (2015). Anhedonia in melancholic and non-melancholic depressive disorders. *Journal of Affective Disorders*.

[8] Riemann D., Berger M., Voderholzer U. (2001). Sleep and depression - Results from psychobiological studies: An overview. *Biological Psychology*.

[9] Pillai V., Kalmbach D. A., Ciesla J. A. (2011). A meta-analysis of electroencephalographic sleep in depression: Evidence for genetic biomarkers. *Biological Psychiatry*.

[10] Baglioni C., Nanovska S., Regen W. (2016). Sleep and mental disorders: A meta-analysis of polysomnographic research. *Psychological Bulletin*.

[11] Kupfer D. J., Foster F. G. (1972). Interval between onset of sleep and rapid-eye-movement sleep as an indicator of depression. *The Lancet*.

[12] Benca R. M., Obermeyer W. H., Thisted R. A., Gillin J. C., Kupfer D. J., Reynolds C. F. (1992). Sleep and psychiatric disorders: a meta-analysis. *Archives of General Psychiatry*.

[13] Asaad T., Sabry W., Rabie M., El-Rassas H. (2016). Polysomnographic characteristics of bipolar hypomanic patients: Comparison with unipolar depressed patients. *Journal of Affective Disorders*.

[14] Spiker D. G., Coble P., Cofsky J., Foster F. G., Kupfer D. J. (1978). EEG sleep and severity of depression. *Biological Psychiatry*.

[15] Jindal R. D., Thase M. E., Fasiczka A. L. (2002). Electroencephalographic sleep profiles in single-episode and recurrent unipolar forms of major depression: II. Comparison during remission. *Biological Psychiatry*.

[16] Guilleminault C., Tilkian A., Dement W. C. (1976). Sleep apnea syndromes. *Annual Review of Medicine*.

[17] Beck A., Beamesderfer A., Pichot P. (1974). Assessment of depression: the depression inventory. *Psychological Measurements in Psychopharmacology: Modern Problems in Pharmacopsychiatry*.

[18] Kemp B., Värri A., Rosa A. C., Nielsen K. D., Gade J. (1992). A simple format for exchange of digitized polygraphic recordings. *Electroencephalography and Clinical Neurophysiology*.

[19] Rechtschaffen A., Kales A. (1968). *A Manual of Standardized Terminology Techniques and Scoring System for Sleep Stages of Human Subjects*.

[20] Winer E. S., Nadorff M. R., Ellis T. E., Allen J. G., Herrera S., Salem T. (2014). Anhedonia predicts suicidal ideation in a large psychiatric inpatient sample. *Psychiatry Research*.

[21] Reynolds C. F., Kupfer D. J., Thase M. E. (1990). Sleep, gender, and depression: An analysis of gender effects on the electroencephalographic sleep of 302 depressed outpatients. *Biological Psychiatry*.

[22] Le Bon O., Staner L., Hoffmann G., Kentos M., Pelc I., Linkowski P. (2001). Shorter REM latency associated with more sleep cycles of a shorter duration in healthy humans. *Psychiatry Research*.

[23] Le Bon O., Hoffmann R., Staner L., Armitage R. (2009). Relationships between the number of ultradian cycles and key sleep variables in outpatients with major depressive disorder. *Psychiatry Research*.

[24] Merica H., Blois R., Bovier P., Gaillard J.-M. (1993). New variables for defining sleep continuity. *Physiology & Behavior*.

[25] Spijker J., Bijl R. V., De Graaf R., Nolen W. A. (2001). Determinants of poor 1-year outcome of DSM-III-R major depression in the general population: Results of the Netherlands Mental Health Survey and Incidence Study (NEMESIS). *Acta Psychiatrica Scandinavica*.

[26] Perils M. L., Giles D. E., Buysse D. J., Thase M. E., Tu X., Kupfer D. J. (1997). Which depressive symptoms are related to which sleep electroencephalographic variables?. *Biological Psychiatry*.

[27] Giles D. E., Roffwarg H. P., Schlesser M. A., Rush A. J. (1986). Which endogenous depressive symptoms relate to REM latency reduction?. *Biological Psychiatry*.

[28] Willner P., Towell A., Sampson D., Sophokleous S., Muscat R. (1987). Reduction of sucrose preference by chronic unpredictable mild stress, and its restoration by a tricyclic antidepressant. *Psychopharmacology*.

[29] Willner P. (1991). Animal models as simulations of depression. *Trends in Pharmacological Sciences*.

[30] Gronli J., Murison R., Bjorvatn B., Sørensen E., Portas C. M., Ursin R. (2004). Chronic mild stress affects sucrose intake and sleep in rats. *Behavioural Brain Research*.

[31] Cheeta S., Ruigt G., Van Proosdij J., Willner P. (1997). Changes in sleep architecture following chronic mild stress. *Biological Psychiatry*.

[32] Moreau J.-L., Scherschlicht R., Jenck F., Martin J. R. (1995). Chronic mild stress-induced anhedonia model of depression: Sleep abnormalities and curative effects of electroshock treatment. *Behavioural Pharmacology*.

[33] Moreau J.-L. (2002). Simulating the anhedonia symptom of depression in animals. *Dialogues in Clinical Neuroscience*.

[34] Wang Z., Yu B., Zhang X. (2014). Correlations between depression behaviors and sleep parameters after repeated corticosterone injections in rats. *Acta Pharmacologica Sinica*.

[35] Yacoubi M. E., Popa D., Martin B. (2012). Genetic association between helpless trait and depression-related phenotypes: Evidence from crossbreeding studies with H/Rouen and NH/Rouen mice. *The International Journal of Neuropsychopharmacology*.

[36] Torterolo P., Scorza C., Lagos P. (2015). Melanin-concentrating hormone (MCH): Role in REM sleep and depression. *Frontiers in Neuroscience*.

[37] Schmidt F. M., Nowak C., Kratzsch J., Sander C., Hegerl U., Schönknecht P. (2015). Dynamics of melanin-concentrating hormone (MCH) serum levels in major depressive disorder during antidepressant treatment. *Journal of Affective Disorders*.

[38] Ansseau M., Kupfer D. J., Reynolds C. F. (1985). Internight variability of REM latency in major depression: Implications for the use of REM latency as a biological correlate. *Biological Psychiatry*.

[39] Ohayon M. M., Carskadon M. A., Guilleminault C., Vitiello M. V. (2004). Meta-analysis of quantitative sleep parameters from childhood to old age in healthy individuals: developing normative sleep values across the human lifespan. *SLEEP*.

[40] Palagini L., Baglioni C., Ciapparelli A., Gemignani A., Riemann D. (2013). REM sleep dysregulation in depression: State of the art. *Sleep Medicine Reviews*.

